# Longitudinal CSF Tumor Cell Enumeration and Mutational Analysis as a Driver for Leptomeningeal Disease Management

**DOI:** 10.3390/cancers17050825

**Published:** 2025-02-27

**Authors:** Arushi Tripathy, Pericles Corkos, Barbara Blouw, Deondra A. Montgomery, Melissa Moore, Marc H. Hedrick, Michael Youssef, Priya U. Kumthekar

**Affiliations:** 1 Department of Neurosurgery, University of Michigan, Ann Arbor, MI 48109, USA; 2 AGEA Biotechnologies, San Diego, CA 92101, USA; perry.corkos@gmail.com; 3 Plus Therapeutics, Austin, TX 78756, USA; 4 College of Human Medicine, Michigan State University, East Lansing, MI 48808, USA; 5 Department of Neurology, University of Southwestern Medical Center, Dallas, TX 75390, USA; 6 Neurology (Neuro-Oncology) and Medicine (Hematology and Oncology), Northwestern Medicine Lou and Jean Malani Brain Tumor Institute, Chicago, IL 60611, USA

**Keywords:** biomarkers, cerebrospinal fluid cytology, cerebrospinal fluid tumor cells, CNSide, leptomeningeal disease

## Abstract

Background: Leptomeningeal disease (LMD) is challenging to diagnose and monitor given the poor sensitivity of current gold-standard diagnostics. Cerebrospinal fluid tumor cells (CSF-TCs) have been studied as a biomarker for disease management because oncogene amplification of the primary, metastatic, and CNS metastatic tumors can be heterogeneous. The CNSide platform enumerates CSF-TCs and analyzes oncogene expression via immunocytochemistry (ICC), fluorescent in situ hybridization (FISH), and next-generation sequencing (NGS). We report the utility of this combined enumerative and mutational testing for LMD diagnosis and disease monitoring. Methods: A multicenter, retrospective analysis of commercially ordered assays from two health systems between January 2020 and July 2023 included 613 tests on 218 individual patients with suspected or confirmed LMD. To date, this is the largest cohort of patients in LMD literature evaluated using CSF-TCs. Results: CSF-TCs were detected in 67% (412/613) of samples. The most analyzed cancer types were breast (*n* = 105) and lung (*n* = 65). In lung cancer, anaplastic lymphoma kinase (ALK) was detected in 14% (17/118), and c-MET was detected in 61% (78/128). In breast cancer, HER2 was detected in 39% (65/168), and estrogen receptor (ER) was detected in 26% (44/168). Sixty-six patients underwent 2+ longitudinal CSF draws; among these, there were 58 flips in oncogene detection over time, and 30% (20/66) of patients had at least one biomarker change in the CSF. Conclusions: Longitudinal combined ICC/FISH/NGS CSF testing demonstrates a wide range in CSF-TC enumeration, which may be correlated with clinical course, and furthermore identifies actionable tumor markers that frequently fluctuate over time. Utilization of this platform would enable timely, personalized LMD-specific chemotherapy.

## Simple Summary

This research focuses on better diagnosing and monitoring of leptomeningeal disease (LMD), a condition hard to detect with current methods. We use a new approach called CNSide, which examines cancer cells in cerebrospinal fluid (CSF) to look for specific genetic markers. We analyzed data from tests on 218 patients, finding that this method detected cancer cells in 67% of cases. By identifying changing tumor markers, doctors can offer more personalized treatments for LMD, which could improve patient outcomes. This research impacts the medical community by demonstrating the feasibility of a more sensitive way to detect and monitor LMD, potentially leading to more effective, targeted therapies.

## 1. Introduction

Leptomeningeal disease (LMD), the infiltration of the pia and arachnoid matter of the CNS by metastatic cancer cells, is a devastating, often late-stage complication of solid and hematogenous malignancies [1]. LMD is diagnosed in 5–10% of patients with cancer; however, the incidence of LMD is likely rising with increasing overall survival among patients with systemic cancer [2,3,4]. Contemporary diagnostics have low sensitivity; thus, LMD is likely grossly underdiagnosed [2,5]. Lumbar punctures (LP) with CSF cytology are less than 60% sensitive, with up to 80% sensitivity with repeated LP [6]. Of patients with solid tumors and positive cytology, MRI abnormalities are only recognized in 40%. Even fewer patients with hematologic malignancies have reliable MRI findings [7].

While prognosis is 4–6 weeks without treatment, overall survival (OS) extends to several months with treatment [8]. Early diagnosis is critical for effective treatment with CNS radiation therapy, systemic chemotherapy with blood–brain barrier penetration, and intrathecal chemotherapy [9]. The challenges in reliable LMD detection severely limit timely treatment initiation, disease monitoring, and patient-specific therapy modification.

Biomarkers, including CSF tumor cells (CSF-TC), tumor DNA (ctDNA), cell-free DNA, RNA, extracellular vesicles, tumor-associated proteins, and specific mutational status, have been increasingly studied as alternative methods for LMD detection [10,11]. CSF ctDNA tests have shown up to 100% sensitivity but have been time-consuming and are currently unavailable for generalized usage [12,13]. CNSide, a commercially offered assay (processed in a College of American Pathologists (CAP)-accredited, Clinical Laboratory Improvement Amendments (CLIA)-certified laboratory), enumerates tumor cells and detects CSF biomarkers using fluorescence in situ hybridization (FISH) or immunocytochemistry (ICC) and next-generation sequencing (NGS). The platform has been validated both in an artificial gradient of TC-augmented CSF as well as among CSF samples obtained from patients with and without LMD diagnosis, demonstrating 92% sensitivity and 95% specificity for LMD [14]. Previous studies comparing cytology and CNSide in smaller cohorts of patients with lung or breast cancer reported that, in up to 38% of CSF samples, CNSide detected tumor cells, but cytology was negative, suggesting that the platform may be more sensitive than the standard of care (SOC) [15,16,17,18,19]. Furthermore, all previous studies found that CNSide identified CSF-TCs in 100% of samples positive for LMD via cytology and/or MRI [15,20,21,22]. CNSide’s multifarious approach and higher sensitivity led to its broad use to rule out LMD in uncertain cases.

Mutational status can differ in the primary, metastatic, and CNS metastatic tumor [16,20], which can affect both chemotherapy choice and prognostication. Due to branched clonal evolution as well as unique therapeutic considerations in treating CNS disease (i.e., blood–brain barrier penetration), a more tailored approach is required in chemotherapy selection among patients with intracranial metastases [21]. In LMD, unlike SOC cytology, combined ICC/FISH/NGS testing can guide therapy choice by detecting actionable mutations, even in cancers in which a particular variant is rare. Previous studies found that this method detected targetable HER2 mutations not only in breast cancer LMD but also in non-small cell lung cancer (NSCLC) and upper gastrointestinal cancer in whom the primary tumor biopsy was negative for HER2 [23]. In breast cancer, the concordance of ER and HER2 status between CSF and systemic metastases was only 60% and 75%, respectively, suggesting that LMD treatment differs from both primary and metastatic cancer therapy [15]. Furthermore, longitudinal CSF-TC FISH analysis demonstrated “flips” in HER2 mutational status, which has actionable consequences for therapeutic management [16].

Longitudinal CSF-TC quantification has previously exhibited utility as an indicator for response to craniospinal irradiation [22]. Longitudinal case series in both NSCLC and breast cancer demonstrated correlation between clinical course and CSF-TC enumeration [16,18]. 

Herein, we describe the large-scale utilization of longitudinal CSF-TC enumeration and FISH/ICC/NGS analysis across multiple sites in the largest cohort of patients in LMD literature evaluated using CSF-TC.

## 2. Materials and Methods

### 2.1. Study Design and Setting

A multicenter, retrospective analysis of commercially ordered CNSide assays between January 2020 and July 2023 was performed. The assay was ordered at the physician’s discretion by 19 physicians from 5 institutions within 2 health systems: (1) The Lou and Jean Malnati Brain Tumor Institute at Northwestern University and (2) The University of Texas Southwestern Medical Center. Data collection was approved by both institutions, also waiving informed consent.

### 2.2. Inclusion Criteria and Protocols

Patients with suspected or confirmed LMD of any cancer histology were included. The CSF collection protocol and time points adhered to the SOC in the diagnosis and monitoring of patients with LMD. CSF was stored in standardized collection tubes and underwent institutional histopathologic and cytologic analysis, and any remaining CSF was submitted for CNSide analysis. Panels corresponding to classically described markers in certain tumor types were available; if no panel was chosen, the clinician could choose up to four biomarkers for analysis in addition to NGS (Supplementary Materials Figure S1).

### 2.3. CNSide Platform Analysis

CSF was aliquoted into CEE-Sure^TM^ collection tubes (Biocept, San Diego, CA, USA) containing a preservative that stabilizes ctDNA and CSF-TCs under ambient conditions for up to 96 h during transport to the testing site. In their CLIA-certified, CAP-accredited laboratory, samples were centrifuged and pelleted. A 10-antibody cocktail was used to capture cells, which were then biotinylated and passed through a streptavidin-coated microfluidic device for immobilization, shown to have superior capture over traditional CSF-TC capture techniques [23]. Cells positive for 4′,6′-diamindino-2-phenylindole (DAPI) and negative for CD45 were identified as CSF-TCs for enumeration. Cells were further analyzed for the presence of tumor-associated cytokeratins as previously described by Mikolajczyk et al. [24].

Immobilized cells were hybridized with ICC probes detecting amplification for PD-L1, ER, and PR and FISH probes for ALK, EGFR, CMET, CMyc, HER2, NTRK1, NTRK3, PTEN, RET, and ROS1. In each sample, up to 100 cells were randomly selected for evaluation. CSF volume (range 2–9 cm^3^) and CSF-TC quantity analyzed varied secondary to clinical factors, including tumor burden and treatment stage.

From the same CSF sample, cell-free total nucleic acids were extracted and used for Switch Blocker^TM^ (following DNA extraction and reverse transcription, RT PCR is followed by Sanger Sequencing for single gene detection) and NGS analysis using Torrent Suite and Ion Reporter with annotation and curation by Oncomine^TM^ Knowledgebase Reporter software (ThermoFisher, Waltham, MA, USA). No clear correlation has been identified between analytes identified on CSF-TCs by ICC/FISH and those identified using cell-free NGS.

## 3. Results

### 3.1. Patient and CSF Collection Characteristics

**Figure 1:**
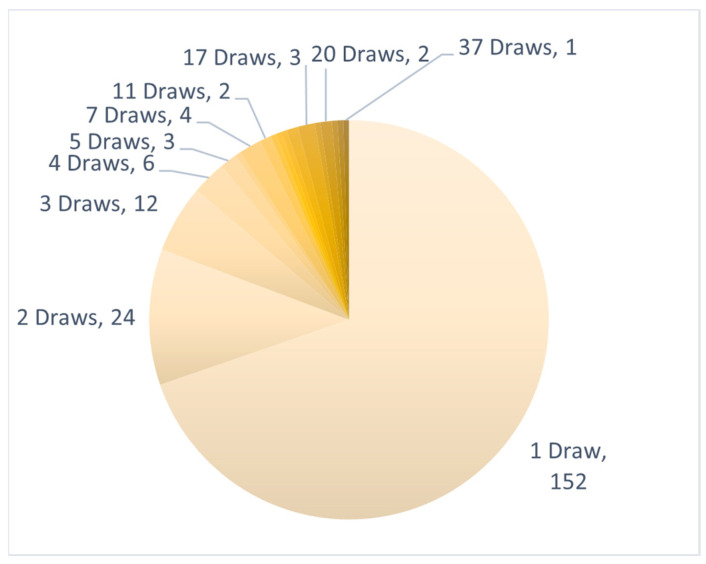
CSF draws per patient: Number of longitudinal CNSide tests ordered for a single patient. Clockwise: from minimum (1) to maximum (37) CSF draws in a single patient.

Six hundred thirteen tests were ordered on 218 individual patients from both health care systems. Sixty-six patients underwent at least two CSF collections, 42 patients underwent at least three CSF collections, and 24 patients underwent five or more CSF collections. The maximum number of CSF draws in a single patient was 37 (Figure 1).

At 74% (162/218), most patients were female; the patients’ age ranged from 19 to 99 years (median = 58) Patients had various primary cancers, including breast (*n* = 105), lung (*n* = 65), GI (*n* = 10), skin (*n* = 8), head and neck (*n* = 4), neuroendocrine (*n* = 3), renal (*n* = 3), pancreatic (*n* = 2), bladder, gynecologic, and hepatic (*n* = 1 each; Table 1). In total, 156 samples were obtained via LP, 280 samples were obtained via Ommaya reservoir access, and 177 samples were not recorded (Table 2).

### 3.2. CSF-TC Enumeration

**Figure 2:**
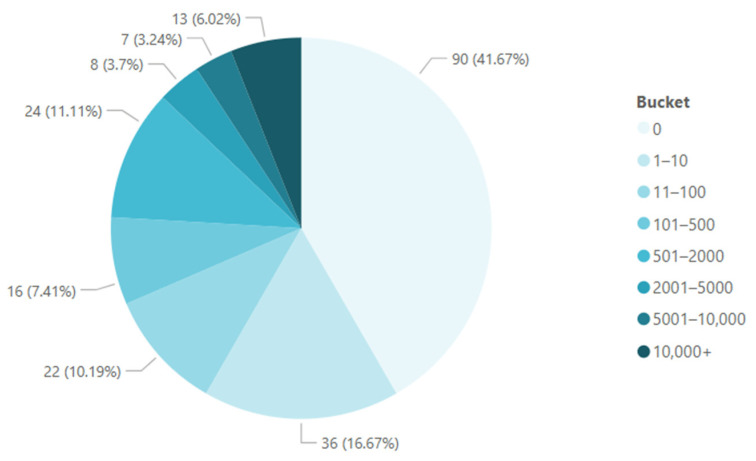
CSF-TC enumeration at primary draws: Clockwise from minimum to maximum, the pie chart represents the range of CSF-TC enumeration values (buckets), and the frequency with which each enumeration value was found is represented in percentages.

CSF-TCs were detected in 55% (120/218) of primary draws, 67% (412/613) of total draws, 66% (195/294) of patients with breast cancer, 76% (175/229) of patients with lung cancer, and 100% of patients with hepatic (7/7) and pancreatic (5/5) cancers (Table 3). Enumeration ranged from zero to more than 10,000 TCs detected per sample (Figure 2). A total of 7.3% of the TCs captured on primary draw were CK− (Table 4).

### 3.3. ICC and FISH Marker Detection

**Figure 3:**
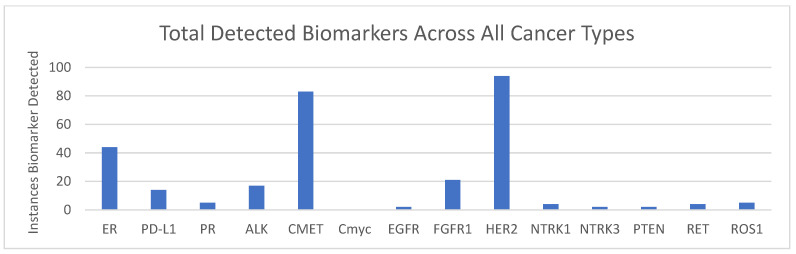
Biomarkers detected across all samples: The total number of case alterations were detected in each biomarker using ICC (ER, PD-L1, and PR) or FISH (remainder of markers) across all samples.

ICC analysis included PD-L1, ER, and PR, and FISH analysis included ALK, EGFR, CMET, CMyc, HER2, NTRK1, NTRK3, PTEN, RET, and ROS1 (Figure 3). Markers were detected in as few as one analyzed cell and have previously been analytically validated by Biocept per CAP guidelines. In patients with lung cancer, ALK was detected in 14% (17/118), CMET was detected in 61% (78/128), HER2 was detected in 73% (16/22), and RET was detected in 4% (4/90). In patients with breast cancer, HER2 was detected in 39% (65/168), FGFR1 was detected in 32% (19/60), ER was detected in 26% (44/168), and PR was detected in 4% (5/120) (Table 5).

Of the 66 patients who underwent two or more CSF draws, 20 (30.3%) had at least one longitudinal change in marker detection (“flip”) among their ordered biomarkers (Table 6).

**Figure 4:**
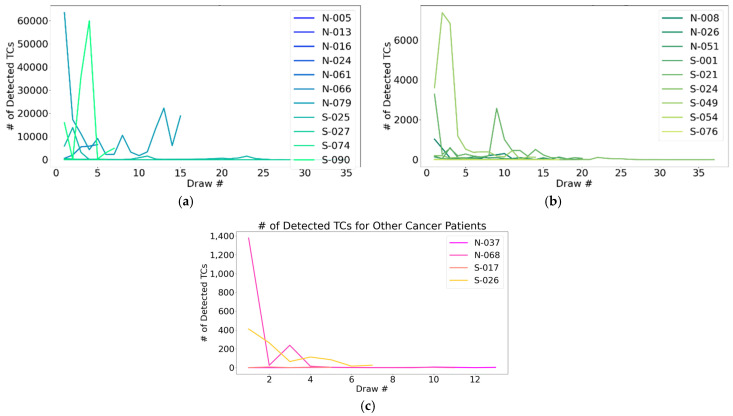
CSF-TC enumeration over longitudinal CSF draws: A representation of the ability to monitor CNS disease over time using detectable changes in CSF-TC enumeration, specifically including (**a**) patients with breast cancer; (**b**) lung cancer; and (**c**) other primary tumors. “Other cancers” included bladder, gastrointestinal, gynecologic, head and neck, hepatic, male genitourinary, neuroendocrine, pancreatic, renal, skin, and miscellaneous.

Among the patients who underwent 5+ longitudinal draws, prominent fluctuations in CSF-TC enumeration were observed, demonstrating an ability to capture variation in LMD over time (Figure 4).

### 3.4. Next-Generation Sequencing (NGS)

NGS detected one or more variants in 39% of patients (144/368). Among the patients with breast or lung cancer, variants were detected in 32% and 55%, respectively (Table 7).

## 4. Discussion

This microfluidic device’s CSF-TC detection rate was previously demonstrated to have 92% sensitivity and 95% specificity among patient CSF samples [14] and up to 100% sensitivity in case series [15,16,17,18], leading clinicians to employ the assay as an LMD rule-out in cases with negative cytology. In our cohort, of the 96 cases of suspected LMD in which initial draws did not detect CSF-TCs, 86 (89.6%) patients did not undergo further testing. This suggests that the assay was used as a final determinant of LMD absence. Given the utilization of this assay in cases of both suspected and confirmed LMD, and especially its utilization as a confirmation of negative cytology, the detection rates in our cohort (67%, 412/613) cannot be compared to sensitivity rates that only include patients with LMD confirmed via cytology and/or imaging. Unfortunately, in this real-world dataset, cytology results were not available for parallel comparison.

### 4.1. Nominating CSF-TC Enumeration as a Prognostic Biomarker

CSF-TC quantification before and after craniospinal irradiation has been shown to be a possible biomarker for progression-free survival (PFS) prognostication [22]. In a sample of nearly 30 patients, CSF-TC density as assessed via microfluidic device capture was found to be significantly associated with survival [25]. In our dataset, we observed highly precise enumeration, with the ability to quantify <10 CSF-TCs up to 10,000 CSF-TCs (Figure 2). Further studies among larger cohorts correlating CSF-TC density with patient outcomes are needed to compartmentalize this spectrum into clinically applicable OS and PFS estimates. Precise CSF-TC density may help clinicians risk-stratify patients with LMD and guide treatment decisions.

### 4.2. Nominating Longitudinal CSF-TC Enumeration as a Marker of Therapy Response

Given its precision in enumeration, other groups have utilized this assay to correlate changes in CSF-TC density with clinical response. Various case series and retrospective analyses across institutions have found that microfluidic CSF-TC enumeration has tracked response to therapy longitudinally [18,25,26]. Though LMD is a uniformly progressive and fatal condition, we identified instances in which subsequent CSF draws were negative for CSF-TCs (Table 3). The change in detection may reflect fluctuation in disease course or response to treatment. While our dataset does not include clinical correlatives, among the patients who underwent 5+ longitudinal draws, we identified prominent fluctuations in CSF-TC enumeration, demonstrating an ability to capture variation in LMD over time (Table 5).

### 4.3. Detection of Mutations Unique to the LMD Tumor Expands Treatment Options

Previous studies have demonstrated discordance of oncogenic driver mutations between primary tumors and solid brain metastases, with clinical trials evaluating the efficacy of various chemotherapeutic agents specifically in CNS metastases [21]. ICC/FISH analysis using this microfluidic device may nominate patients with LMD for participation in these trials.

In our cohort, among the patients with lung cancer, ALK was detected in 14% (17/118), and c-MET was detected in 61% (78/128). These are relatively uncommon oncogenic drivers in NSCLC, comprising just 4–5% and 3–4%, respectively [26]. Five tyrosine kinase inhibitors have been approved by the FDA for the treatment of ALK+ NSCLC, and at least two have been approved for c-MET alterations [27]. Furthermore, RET was detected in 4% (4/90), ROS1 was detected in 3.4% (4/117), and HER2 was detected in 73% (16/22)—all are also targetable with approved pharmaceuticals.

In the patients with breast cancer, HER2 was detected in 39% (65/168), FGFR1 was detected in 32% (19/60), ER was detected in 26% (44/168), and PR was detected in 4% (5/120). In another study by our group, for patients in whom primary tumor mutational status was available for comparison, a difference in HER2 status between primary tumor and CSF was found in 10/26 (38%) of patients [16]. Therefore, in a significant subset of patients, the efficacy of anti-HER2 therapy on LMD likely differs from the primary tumor. A variety of ER-targeted endocrine therapies are being tested in clinical trials [28]; ER detection in CSF-TCs may help patients with LMD meet the criteria to enroll. LMD tumor mutations uncommon to and/or discordant with the primary tumor could considerably change chemotherapeutic agent choice.

### 4.4. Repeat Analysis Detects Changes (“Flips”) in Targetable Mutations

While previous studies in smaller cohorts have found 100% concordance of single-time point CSF-CT mutational analysis and tissue NGS [17], in our cohort, ICC and FISH probe detection status was found to change over the course of multiple draws. Sixty-six patients in our cohort underwent 2+ CSF draws; and 24 underwent 5+. There were 13 ICC detection flips (seven acquired mutations) and 45 FISH probe detection flips (26 acquired mutations). Twenty of 66 patients (30.3%) had at least one flip in their ordered biomarkers. Most prominently, 12 patients showcased a flip in HER2 positivity over their disease course via CNSide testing, as previously noted [16]; however, flips were also identified in CMET in eight patients, FGFR1 in seven patients, ALK in five patients, ROS1 in four patients, and RET in three patients (Table 6). Changes in mutational status indicated time points at which chemotherapies could have been altered to best personalize treatment to the LMD tumor. Flips in targetable markers over time could explain acquired resistance to chemotherapies (e.g., due to loss of targetable mutation expression) or could nominate new chemotherapies targeting acquired mutations for effective treatment. While our dataset does not include associated information regarding treatment regimen modifications during the course of CNSide monitoring, our group previously noted that, in at least one patient with a HER2-negative primary tumor and HER2 positivity on CNSide testing, anti-HER2 therapy was initiated and produced a clinical response [16]. Retrospective correlation between treatment regimen changes and CSF-TC mutational analysis is of limited utility due to a multitude of additional factors that are part of therapeutic decision-making in patients with LMD, including therapy tolerance, systemic disease status, and, especially in patients with LMD who are often in the very late stages of stage 4 systemic cancer, transition towards palliative care and hospice as an indication for therapy termination. Therefore, prospective assessment using CNSide to help guide clinical decision-making will present a more complete picture of its true utility.

### 4.5. CSF-TC NGS May Optimize Therapeutic Regimens

In our cohort, NGS analysis detected at least one variant in the CSF samples from the patients with breast cancer 32% of the time and the patients with lung cancer 55% of the time. Comparatively, primary breast cancer biopsies have been found to carry variants in 62% of cases [29], and serum samples in patients with NSCLC were found to demonstrate variants in 81.5% of cases [30]. This difference in detection rates in the primary tumor vs. CSF NGS may reflect variability in the genetic profile in the primary and CNS/LMD tumor, as previously demonstrated [20]. Identification of genetic variants present in CSF could narrow the therapeutic regimen to drugs that target the LMD tumor specifically or could predict whether chosen chemotherapeutic agents will be effective on the CNS disease.

### 4.6. CNSide Analysis Captures Cells in All EMT States

Previous CSF-TC capturing technologies have relied upon anti-epithelial cell adhesion molecule (EpCAM) antibodies [31], with TC detection using cytokeratin (CK) antibodies, limiting detection to CSF-TCs expressing EpCAMs. However, it has been well described that metastatic cells undergo epithelial-to-mesenchymal transition (EMT) and express varying to even undetectable amounts of EpCAM, implying that standard CSF-TC detection fails to capture a proportion of tumor cells. CellSearch^TM^, which uses anti-EpCAM antibodies for circulating tumor cell (CTC) detection, is currently the only FDA-approved CTC detection in blood. It has been shown that CellSearch^TM^ tumor cell detection misses CTC detection in blood in nearly half of patients compared with detection using a more varied antibody probe selection [32]. The technique utilized by CNSide has been shown to better capture tumor cells in various stages of EMT [23]. This superior capture may explain its improved sensitivity over standard cytology.

While a previous pilot study investigating the clinical relevance of CK-CTCs in blood did not find an association between CK-CTCs and poor outcomes [32], the clinical significance of CK− CSF-TCs has not been defined. CNSide is unique compared to previous cytologic methods of CSF-TC detection in its ability to capture CK− cells [23]. In our dataset, 7.3% of the CSF-TCs captured on primary draw and 2.6% of subsequent draws were CK− (Table 4). These are CSF-TCs that would have otherwise been missed in traditional methods of CSF-TC capture and could have produced a false negative result in some samples. The clinical significance of CK− CSF-TCs has not yet been validated, at least in part due to failure of prior methods to capture these cells. Future clinical series will be necessary to specifically evaluate the clinical relevance of the presence and percentage of CK− cells present in CSF.

Notably, we found that a significant percentage (37%) of primary draw CSF-TCs in the patients with lung cancer LMD were CK− and likely to be overlooked using traditional screening methods. Interestingly, only 7% of subsequent draw CSF-TCs in the patients with lung cancer were CK− (Table 4). We may postulate that treatment of NSCLC or the length of the disease course may affect CK positivity of CSF-TCs. Further longitudinal analysis of lung cancer CSF-TCs is needed to study the change in surface markers and EMT over the disease course.

### 4.7. Redefining LMD Diagnosis: From Cytology to CSF-TC Enumeration

The ability to detect minimal numbers of CSF-TCs presents an interesting diagnostic question: on initial draw in a patient with intracranial metastatic disease, do small numbers of CSF-TCs capture early LMD or are they sloughed cells from brain metastases? Prospective studies are necessary to define an enumeration threshold for definitive LMD diagnosis and its sensitivity versus gold-standard cytology.

### 4.8. LP vs. Ommaya CSF Draw for CSF-TC Detection

Only 11% (22/218) of initial samples vs. 65% (256/395) of subsequent samples were collected via Ommaya reservoir access. On the flip side, at least 56% (123/218) of initial vs. only 8% (33/395) of subsequent samples were obtained via LP. Detection rates also varied by modality of access: a total of 74% (207/279) of Ommaya access vs. 47% (73/156) of LPs detected CSF-TCs.

The majority of the initial samples in whom the access method was recorded were obtained via LP (123/218, 56%), while the majority of subsequent samples were obtained via Ommaya reservoir access (256/395, 65%) (Table 2). These data could be attributed to increased testing in patients with Ommaya reservoir due to ease of access or could indicate preferential Ommaya placement and longitudinal testing among patients with severe or unequivocal LMD.

Detection rates also varied by the modality of access: a total of 74% (207/279) of Ommaya access vs. 47% (73/156) of LPs detected CSF-TCs. This difference could be attributable to improved detection with Ommaya (cranial) CSF collection or due to a higher pretest probability of CNS cancer in patients in whom an Ommaya reservoir is placed. Unfortunately, our dataset does not include comparative LP and Ommaya results in the same patient at the same time points. Prospective CSF collection using these parallel methods is indicated to determine whether CSF from LP or intracranial CSF is more accurate in detecting LMD.

### 4.9. Limitations

Our data are limited by their retrospective nature and lack of clinical correlation (e.g., cytology results), which will be partially addressed by the pending results of the FORESEE trial (NCT05414123). The trial aimed to characterize CNSide’s utility in patients with breast cancer and NSCLC LMD, recruiting 40 patients before suspension due to the financial insolvency of the company. Despite the benefits, the cost and availability of combined CSF-TC enumeration and mutational analysis remain concerns for widespread implementation. There is a critical need for the development and availability of high-sensitivity CSF-TC testing, and further studies are recommended to evaluate the long-term benefits of these methods in standard LMD diagnostic protocols.

## 5. Conclusions

Compared to cytology, MR imaging, and clinical assessment, microfluidic CSF-TC enumeration is more sensitive for tumor cell detection and may, therefore, be a future gold standard for LMD diagnosis. Its superior ability to detect CSF-TCs may catalyze treatment initiation, which has been shown to prolong survival [8,25]. Furthermore, it detects LMD mutations discordant to the primary tumor, widening therapeutic options [16]. Herein, we demonstrate that, longitudinally, the assay offers the ability to quantify LMD over time and monitor changes in the expression of multiple targetable mutations. In some cases, the CNSide assay identified in LMD a higher incidence of mutations that are rare in the primary tumor.

Our data suggest CSF-TC analysis platforms are highly sought after for diagnosis and to inform longitudinal treatment decisions by clinicians treating patients with metastatic brain tumors. Their clinical implementation could improve outcomes by enabling earlier intervention with targeted therapies.

## Figures and Tables

**Table 1:** Samples by primary tumor type: The 13 primary tumor categories from which CSF samples were analyzed.

Primary Tumor Type	Number of Samples	Number of Patients
Bladder	1	1
Breast	294	105
GI	24	10
Gynecologic	1	2
Head and Neck	6	4
Hepatic	7	1
Lung	229	65
Male GU	3	2
Miscellaneous	24	12
Neuroendocrine	3	3
Pancreatic	5	2
Renal	3	3
Skin	13	8
Total	613	218

Abbreviations: GI, Gastrointestinal; GU, Genitourinary.

**Table 2:** Modalities of CSF draw organized by primary and subsequent CSF draws and detection of CSF-TCs.

	Ommaya	Lumbar Puncture	Not Recorded	Total
Primary	23	123	72	218
Subsequent	256	33	106	395
Detected	207	73	122	402
Not detected	71	83	55	209
NGS only	1	0	1	2
Total	279	156	178	613

**Table 3:** CSF-TC enumeration detection of CK+ and CK− cells at primary and subsequent draws. For each primary tumor histologic type, detection of CSF-TCs is reported in a binary manner (CSF-TCs either present—detected, or absent—not detected). The “miscellaneous” category includes bladder, gynecologic, head and neck, hepatic, male genitourinary, neuroendocrine, pancreatic, and renal.

Primary Tumor Type	Primary Detected	Primary Not Detected	Subsequent Detected	Subsequent Not Detected	Total Detected	Total Not Detected
Breast	62	43	132	56	194	99
Gastrointestinal	1	9	7	7	8	16
Hepatic	1	0	6	0	7	0
Lung	46	19	129	35	175	54
Miscellaneous	7	21	13	4	20	25
Skin	3	4	4	1	7	5
Total	120	96	291	103	411	198

**Table 4:** CK detection by primary tumor type. For each primary tumor type, CSF-TCs that were CK+ and CK− were quantified at primary and subsequent CSF draws. The “miscellaneous” category includes gynecologic, head and neck, hepatic, male genitourinary, neuroendocrine, pancreatic, and renal.

Primary Tumor Type	Number Primary Samples	Primary CK+ %	Primary CK− %	Number Subsequent Samples	Subsequent CK+ %	Subsequent CK− %
Bladder	1	0	0	0	0	0
Breast	105	98	2	36	99	1
Gastrointestinal	10	98	2	3	100	0
Hepatic	1	94	6	1	80	20
Lung	65	63	37	18	93	7
Miscellaneous	12	100	0	2	91	9
Skin	7	32	68	2	0	100
Total	216	93	7	66	97	3

**Table 5:** Biomarker detection via ICC (ER, PD-L1, and PR) or FISH (remainder of markers) by primary tumor type: The number of specific biomarkers detected in breast, lung, and other cancers. “Other” cancers included bladder, gastrointestinal, gynecologic, head and neck, hepatic, male genitourinary, neuroendocrine, pancreatic, renal, skin, and miscellaneous.

	Lung		Breast		Other	
Biomarker	Detected	Not Detected	Detected	Not Detected	Detected	Not Detected
ER	0	0	44	124	0	1
PR	0	2	5	115	0	0
PD-L1	7	157	7	157	0	39
ALK	17	101	0	1	0	11
EGFR	0	2	1	0	1	4
CMET	78	50	0	1	5	3
C-Myc	0	0	0	0	0	1
FGFR1	0	1	19	41	2	1
HER2	16	6	65	103	13	8
NTRK1	1	102	2	131	1	22
NTRK3	0	89	2	92	0	15
PTEN	0	1	0	0	2	4
RET	4	86	0	0	0	7
ROS1	4	113	0	1	1	8

**Table 6:** Flips in biomarker detection over serial CSF testing: Biomarker detections via ICC/FISH for serial CSF draws showed instances in which biomarker detection appeared or disappeared on subsequent draws.

			Flips	
Probe	Number of Patient Samples with >2 Tests	Number of Patients with a Flip in FISH/ICC Biomarker Results	Not Detected -> Detected	Detected -> Not Detected
ER	25	6	2	4
PD-L1	46	7	5	2
PR	16	0	0	0
ALK	14	5	2	3
CMET	17	8	4	4
Cmyc	0	0	0	0
EGFR	1	1	0	1
FGFR1	13	7	5	2
HER2	32	12	5	7
NTRK1	38	3	2	1
NTRK3	30	1	0	1
PTEN	1	1	1	0
RET	12	3	3	0
ROS1	13	4	4	0
Grand total	258	58	33	25

**Table 7:** NGS variant detection by tumor type.

Primary Tumor Type	Primary Detected	Primary Not Detected	Subsequent Detected	Subsequent Not Detected	Total Detected	Total Not Detected
Bladder	0	1	0	0	0	1
Breast	26	42	31	78	57	120
Gastrointestinal	2	6	2	10	4	16
Gynecologic	0	1	0	0	0	1
Head and neck	1	0	0	0	1	0
Hepatic	0	0	0	0	0	0
Lung	25	20	46	37	71	57
Male genitourinary	0	2	0	0	0	2
Miscellaneous	1	8	0	10	1	18
Neuroendocrine	0	2	0	0	0	2
Pancreatic	2	0	2	1	4	1
Renal	0	2	0	0	0	2
Skin	2	4	4	0	6	4
Total	59	88	85	136	144	224

Detection of at least one genomic variant via NGS separated by CSF draw instance.

## Data Availability

The dataset is available on request from the authors.

## Supplementary Materials

The following supporting information can be downloaded at: https://www.mdpi.com/article/10.3390/cancers17050825/s1, Figure S1: BioCept CNSide Requisition Form.

## Abbreviations

The following abbreviations are used in this manuscript:

ALK	Anaplastic Lymphoma Kinase
CAP	College of American Pathologists
CLIA	Clinical Laboratory Improvement Amendments
CSF	Cerebrospinal Fluid
CSF-TC	Cerebrospinal Fluid Tumor Cells
CTC	Circulating Tumor Cells
cfDNA	Cell-Free DNA
ctDNA	Tumor DNA
DAPI	diamindino-2-phenylindole
EMT	Epithelial-to-Mesenchymal Transition
FISH	Fluorescent in situ hybridization
ICC	Immunocytochemistry
LP	Lumbar Punctures
NGS	Next-Generation Sequencing
NSCLC	Non-Small Cell Lung Cancer
OS	Overall Survival
PFS	Progression-Free Survival
SOC	Standard of Care
