# Supplementary material for: Longitudinal CSF Tumor Cell Enumeration and Mutational Analysis as a Driver for Leptomeningeal Disease Management
Source: Cancers (Basel). 2025 Feb 27;17(5):825. doi: 10.3390/cancers17050825 (PMC11899081; doi:10.3390/cancers17050825)
Supplement: Supplementary file 1 [file cancers-17-00825-s001.zip › cancers-3435883-supplementary.pdf]

Supplementary materials

Cerebrospinal fluid tumor cell enumeration and genomic mutational analysis improves diagnosis and disease management in patients with leptomeningeal disease

Arushi Tripathy[1*], Pericles Corkos[2], Barbara Blouw[3], Deondra A. Montgomery[4], Melissa Moore[3], Marc H. Hedrick[3], Michael Youssef[5], Priya U. Kumthekar[6]

***Corresponding author:** Arushi Tripathy, MD. ORCID: 0000-0002-5635-6036. *Email:* tripatha@med.umich.edu

Fig. S1 BioCept CNSide requisition form. A representative requisition form available to physicians ordering the CNSide platform assay, demonstrating choices in biomarker panels

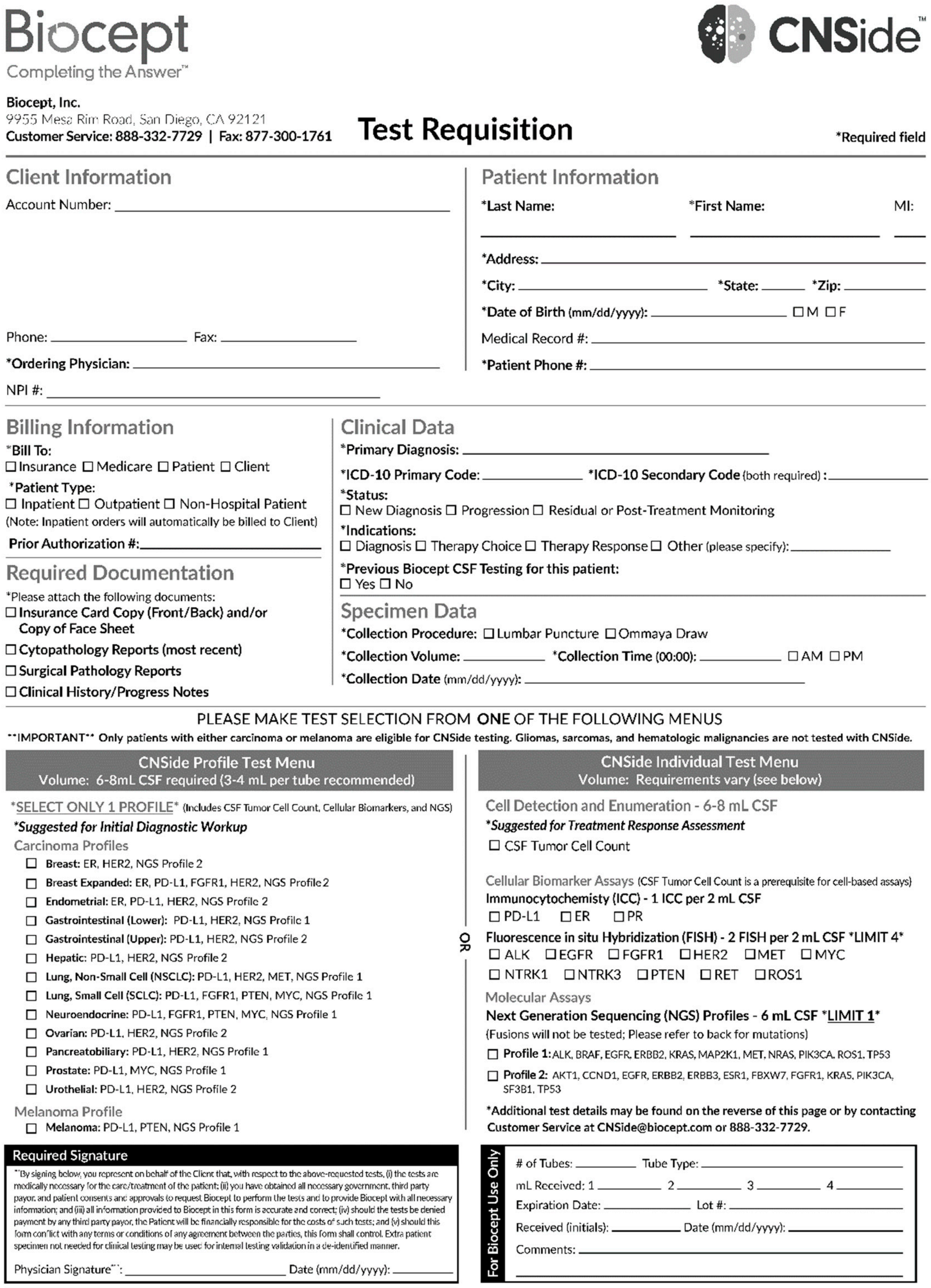

Biocept
Completing the Answer™

CNSide™

Biocept, Inc.
9955 Mesa Rim Road, San Diego, CA 92121
Customer Service: 888-332-7729 | Fax: 877-300-1761

Test Requisition

*Required field

Client Information

Account Number: ______

Phone: ______ Fax: ______

*Ordering Physician: ______

NPI #: ______

Patient Information

*Last Name: ______ *First Name: ______ MI: ______

*Address: ______

*City: ______ *State: ______ *Zip: ______

*Date of Birth (mm/dd/yyyy): ______ ☐ M ☐ F

Medical Record #: ______

*Patient Phone #: ______

Billing Information

*Bill To:
☐ Insurance ☐ Medicare ☐ Patient ☐ Client

*Patient Type:
☐ Inpatient ☐ Outpatient ☐ Non-Hospital Patient
(Note: Inpatient orders will automatically be billed to Client)

Prior Authorization #: ______

Required Documentation

*Please attach the following documents:
☐ Insurance Card Copy (Front/Back) and/or Copy of Face Sheet
☐ Cytopathology Reports (most recent)
☐ Surgical Pathology Reports
☐ Clinical History/Progress Notes

Clinical Data

*Primary Diagnosis: ______

*ICD-10 Primary Code: ______ *ICD-10 Secondary Code (both required): ______

*Status:
☐ New Diagnosis ☐ Progression ☐ Residual or Post-Treatment Monitoring

*Indications:
☐ Diagnosis ☐ Therapy Choice ☐ Therapy Response ☐ Other (please specify): ______

*Previous Biocept CSF Testing for this patient:
☐ Yes ☐ No

Specimen Data

*Collection Procedure: ☐ Lumbar Puncture ☐ Ommaya Draw

*Collection Volume: ______ *Collection Time (00:00): ______ ☐ AM ☐ PM

*Collection Date (mm/dd/yyyy): ______

PLEASE MAKE TEST SELECTION FROM **ONE** OF THE FOLLOWING MENUS

IMPORTANT Only patients with either carcinoma or melanoma are eligible for CNSide testing. Gliomas, sarcomas, and hematologic malignancies are not tested with CNSide.

CNSide Profile Test Menu
Volume: 6-8mL CSF required (3-4 mL per tube recommended)

SELECT ONLY 1 PROFILE (Includes CSF Tumor Cell Count, Cellular Biomarkers, and NGS)

Suggested for Initial Diagnostic Workup

Carcinoma Profiles

- ☐ **Breast:** ER, HER2, NGS Profile 2
- ☐ **Breast Expanded:** ER, PD-L1, FGFR1, HER2, NGS Profile 2
- ☐ **Endometrial:** ER, PD-L1, HER2, NGS Profile 2
- ☐ **Gastrointestinal (Lower):** PD-L1, HER2, NGS Profile 1
- ☐ **Gastrointestinal (Upper):** PD-L1, HER2, NGS Profile 2
- ☐ **Hepatic:** PD-L1, HER2, NGS Profile 2
- ☐ **Lung, Non-Small Cell (NSCLC):** PD-L1, HER2, MET, NGS Profile 1
- ☐ **Lung, Small Cell (SCLC):** PD-L1, FGFR1, PTEN, MYC, NGS Profile 1
- ☐ **Neuroendocrine:** PD-L1, FGFR1, PTEN, MYC, NGS Profile 1
- ☐ **Ovarian:** PD-L1, HER2, NGS Profile 2
- ☐ **Pancreatobiliary:** PD-L1, HER2, NGS Profile 1
- ☐ **Prostate:** PD-L1, MYC, NGS Profile 1
- ☐ **Urothelial:** PD-L1, HER2, NGS Profile 2

Melanoma Profile

- ☐ **Melanoma:** PD-L1, PTEN, NGS Profile 1

OR

CNSide Individual Test Menu
Volume: Requirements vary (see below)

Cell Detection and Enumeration - 6-8 mL CSF

Suggested for Treatment Response Assessment

☐ CSF Tumor Cell Count

Cellular Biomarker Assays (CSF Tumor Cell Count is a prerequisite for cell-based assays)

Immunocytochemistry (ICC) - 1 ICC per 2 mL CSF
☐ PD-L1 ☐ ER ☐ PR

Fluorescence in situ Hybridization (FISH) - 2 FISH per 2 mL CSF *LIMIT 4*
☐ ALK ☐ EGFR ☐ FGFR1 ☐ HER2 ☐ MET ☐ MYC
☐ NTRK1 ☐ NTRK3 ☐ PTEN ☐ RET ☐ ROS1

Molecular Assays

Next Generation Sequencing (NGS) Profiles - 6 mL CSF *LIMIT 1*
(Fusions will not be tested; Please refer to back for mutations)

☐ **Profile 1:** ALK, BRAF, EGFR, ERBB2, KRAS, MAP2K1, MET, NRAS, PIK3CA, ROS1, TP53

☐ **Profile 2:** AKT1, CCND1, EGFR, ERBB2, ERBB3, ESR1, FBXW7, FGFR1, KRAS, PIK3CA, SF3B1, TP53

***Additional test details may be found on the reverse of this page or by contacting Customer Service at CNSide@biocept.com or 888-332-7729.**

Required Signature

**By signing below, you represent on behalf of the Client that, with respect to the above-requested tests, (i) the tests are medically necessary for the care/treatment of the patient; (ii) you have obtained all necessary government, third party payor, and patient consents and approvals to request Biocept to perform the tests and to provide Biocept with all necessary information; and (iii) all information provided to Biocept in this form is accurate and correct; (iv) should the tests be denied payment by any third party payor, the Patient will be financially responsible for the costs of such tests; and (v) should this form conflict with any terms or conditions of any agreement between the parties, this form shall control. Extra patient specimen not needed for clinical testing may be used for internal testing validation in a de-identified manner.

Physician Signature**: ______ Date (mm/dd/yyyy): ______

For Biocept Use Only

# of Tubes: ______ Tube Type: ______

mL Received: 1 ______ 2 ______ 3 ______ 4 ______

Expiration Date: ______ Lot #: ______

Received (initials): ______ Date (mm/dd/yyyy): ______

Comments: ______
